# Dietary Habits and Lifestyle During Coronavirus Pandemic Lockdown: Experience From Lebanon

**DOI:** 10.3389/fnut.2021.730425

**Published:** 2021-08-30

**Authors:** Leila Cheikh Ismail, Mona Hashim, Maysm N. Mohamad, Hussein Hassan, Abir Ajab, Lily Stojanovska, Amjad H. Jarrar, Hayder Hasan, Dima O. Abu Jamous, Sheima T. Saleh, Rameez Al Daour, Tareq M. Osaili, Ayesha S. Al Dhaheri

**Affiliations:** ^1^Clinical Nutrition and Dietetics Department, College of Health Sciences, University of Sharjah, Sharjah, United Arab Emirates; ^2^Nuffield Department of Women's & Reproductive Health, University of Oxford, Oxford, United Kingdom; ^3^Department of Nutrition and Health, College of Medicine and Health Sciences, United Arab Emirates University, Al Ain, United Arab Emirates; ^4^Department of Natural Sciences, School of Arts and Sciences, Lebanese American University, Beirut, Lebanon; ^5^Institute for Health and Sport, Victoria University, Melbourne, VIC, Australia; ^6^Research Institute for Medical and Health Sciences, University of Sharjah, Sharjah, United Arab Emirates; ^7^Department of Nutrition and Food Technology, Faculty of Agriculture, Jordan University of Science and Technology, Irbid, Jordan

**Keywords:** COVID-19, eating habits, food security, Lebanon, lifestyle behaviors

## Abstract

This study aimed to examine the impact of quarantine on eating habits and lifestyle behaviors among the Lebanese adult population. A cross-sectional study was conducted using an online questionnaire designed on Google Forms between 3 June and 28 June 2020. The survey questions were adapted from the Short Food Frequency Questionnaire, the International Physical Activity Questionnaire Short Form, and the second version of the Copenhagen Psychosocial Questionnaire. A total of 2,507 adults completed the questionnaire. During the lockdown, 32.8% claimed weight gain, 44.7% did not eat fruits daily, 35.3% did not eat vegetables on daily basis, and 72.9% reported drinking less than eight cups of water per day. Moreover, there was a significant increase in the number of meals consumed per day, consumption of homemade meals, sedentary time, stress, and sleeping disturbances during the pandemic compared to before the pandemic (all *p* < 0.001). However, there was a significant decrease in physical activity engagement, sleep quality, and energy level during the lockdown compared to before the pandemic (all *p* < 0.001). The study highlights that the COVID-19 lockdown was associated with unfavorable changes in dietary habits and lifestyle behaviors in Lebanon. Sleep and mental health were also negatively impacted by the pandemic.

## Introduction

Coronavirus Disease (COVID-19) is a global public health concern caused by the novel Severe Acute Respiratory Syndrome Coronavirus 2 (SARS-CoV-2) (1). The first reported case of COVID-19 was in Wuhan, China in December 2019, since the virus has spread sequentially and massively to almost every country in the world (2). The World Health Organization (WHO) on March 11, 2020, announced the coronavirus as a pandemic and declared it a “health emergency” after causing more than 111,828 confirmed deaths (3). The outbreak of the pandemic has led to unprecedented health, economic, and environmental crises (4). It has also overwhelmed the healthcare systems and negatively impacted education, mental health, physical activity, and social life (5–7).

The lack of targeted treatment for the COVID-19 has necessitated countries around the globe to adopt policies of varying degrees to control the situation ranging from social distancing in public spaces to stay-at-home orders (8). However, several countries opted for strict lockdown measures to flatten the curve representing daily new cases of the infection (9). The lockdown measures included the closure of recreational places (sports facilities), restaurants, cafes, businesses, and services including schools, universities, nurseries, and merchandise shops except for essential places (10). These strict measures have imposed shifts to digital education and the majority of the population were asked to work from home (telework) with many becoming unemployed (11).

Lebanon is a middle-income country in the Middle East with an unstable economic and political situation and hosts over 1.5 million refugees (12). The first COVID-19 case in Lebanon was confirmed on 21 February 2020 (13), and the number of infected individuals has continued to rise exponentially since. The first death case from COVID-19 was recorded on 10 March 2020 (14). The Lebanese government enacted several measures to control the situation, including general mobilization banning large gatherings, closure of air and land borders, initiation of telework and distance learning, activation of telehealth activities, and closure of non-essential public places. As of 25 May 2021, there were 538, 218 confirmed cases in Lebanon and 7,670 deaths in total (15).

This unprecedented situation has led to the disruption of daily routines and changed trends of nutritional behaviors worldwide (16, 17). One of the major concerns during the COVID-19 pandemic is malnutrition caused by either over-nutrition, under-nutrition, or micronutrient deficiencies (18). With the COVID-19 pandemic, food security, in particular food access and utilization, was affected. At the community level, access to food was limited leading to lower purchases of fresh fruits in favor of canned food and ultra-processed foods rich in fat, sodium, and sugar (19). However, at the individual level, choices related to proper food utilization were still made available (20). Besides, the substantial changes in lifestyle due to containment lead to sedentary behaviors and alterations in sleeping, smoking, and drinking patterns (21, 22). It has been reported that changes in eating patterns and lifestyle due to confinement were steering toward a health-compromising direction (23), even though, maintaining a healthy balanced and diversified diet may play a profound role in maintaining a proper immune response during the “pandemic-associated worldwide quarantine” (24). In Lebanon, reports before the COVID-19 pandemic, disclosed a high prevalence of non-communicable diseases (NCDs) including cardiovascular diseases, diabetes, cancer, and chronic respiratory diseases (25). These health risks were more predominant in the urban population (26). Coexisting to this, there was an increased occurrence of physical inactivity, a modifiable (NCD) risk factor among the Lebanese population (27). Furthermore, the Food and Agriculture Organization (FAO) indicated recently that acute hunger is set to soar in over 20 countries, including Lebanon in the coming months (28). In addition, according to United Nations Economic and Social Commission for Western Asia (ESCWA), more than 50 percent of the population in Lebanon were at risk of failing to access basic food needs by the end of 2020 (29).

Concurrently with the COVID-19 pandemic, starting October 2019 Lebanon has been witnessing an outbreak of a series of civil protests all over the six governorates of Lebanon and a dire economic situation, especially following the August 4, 2020, chemical explosion in Beirut, exacerbating distress levels amongst many of the Lebanese population. To date, still alarming rates of detected coronavirus cases are reported, and the health system failed to concur with the next effortful “wave” of the pandemic. Given these challenges, this study aims to examine the impact of quarantine on eating habits and lifestyle behaviors among the Lebanese adult population.

## Materials and Methods

### Study Design and Participants

This cross-sectional, online survey was conducted in Lebanon between 3 June 2020 and 28 June 2020. The survey followed the same protocol of the cross-sectional, online survey conducted in the Greater Middle East region between 15 April 2020 and 29 April 2020 (17) and in the United Arab Emirates during April and May 2020 (21). A web link to the electronic questionnaire was distributed using e-mail invitations and social media platforms e.g., LinkedIn™, Facebook™, and WhatsApp™. The introductory page of the survey included information about the study, ethical information for the participants, and the option to choose the desired language. Consenting participants proceeded to complete and submit their responses. Only responses with a 100% completion rate were saved into the system of Google Forms. Participants were not rewarded for taking part in the study and all data was collected anonymously. The study was conducted according to the principles embodied in the Declaration of Helsinki (30) and following the ethical code for internet and social media research (31). The protocol of the study was approved by the Research Ethics Committee at the University of Sharjah (REC-20-04-25-02) and the Social Sciences Research Ethics Committee at United Arab of Emirates University (ERS_2020_6106).

In the current study, adults aged 18 years and older were recruited using the convenience and snowball sampling method. To minimize selection bias of snowball sampling, each participant was asked to refer a maximum of three individuals from different households and one per age group (young adults, older adults, elderly). The sample was drawn from the six governorates in Lebanon and recruitment efforts targeted a final sample size of 2,500 participants with a distribution proportional to that of each governorate's adult population, as estimated by the Central Administration of Statistics in Lebanon (32). Mount Lebanon had the highest number of participants (39%), followed by North (20%), South (18%), Bekaa (13%), and Beirut (10%).

### Survey Questionnaire

A structured self-administered web-based questionnaire was administered using Google Document Forms in Arabic, English, and French languages and was hosted via a unique Uniform Resource Locator (URL). The survey was developed, reviewed, and piloted by a group of multidisciplinary scientists at the University of Sharjah (United Arab Emirates), and the United Arab Emirates University (United Arab Emirates). Questions were adapted from the Short Food Frequency Questionnaire (FFQ) (33), the International Physical Activity Questionnaire Short Form (IPAQ-SF) (34), and the second version of the Copenhagen Psychosocial Questionnaire (COPSOQ-II) (35). The design and conduct of the study protocol have been previously described (17, 21).

The questionnaire was divided into seven sections and it included 39 questions in total: (i) personal data (12 questions: age, gender, marital status, number of children the participant has, level of education, employment status, work or study from home, weight, height, change in body weight during the pandemic, perceived health status, and governorate of residence); (ii) source of information (two questions: source of health and nutrition related information); (iii) dietary habits (seven questions: type of most consumed meals, frequency of meals, eating breakfast, skipping meals, reasons for skipping meals, water consumption, and frequency of consumption of certain foods); (iv) shopping (six questions: preparing a grocery list, stocking up on foods, online grocery shopping, reasons for shopping online, reading food labels, and sanitizing or cleaning groceries); (v) physical activity (four questions: frequency of exercising, frequency of household chores, screen time for work or study, and screen time for entertainment); (vi) stress (four questions: physical exhaustion, emotional exhaustion, irritability, and tension); (vii) sleep (four questions: duration of sleep, quality of sleep, having sleep disturbances, and perceived energy level). The full version of the questionnaire is available as a Supplementary File.

Questions related to dietary habits, physical activity, stress, and sleep were asked once regarding the period before the pandemic and another regarding the period during the pandemic. Due to the sudden nature of the COVID-19 outbreak, and because previous information is not available, the cross-sectional design was used to evaluate the effect of the pandemic on highly modifiable factors including dietary habits and lifestyle behaviors.

### Statistical Analysis

Descriptive statistics for the sociodemographic characteristics were reported as counts and percentages. A McNemar test was employed to investigate the difference between categorical variables before and during the COVID-19 pandemic. A Chi-square (χ^2^) test was used to determine the association between categorical variables. BMI was calculated as weight (kilograms)/height^2^ (meters). Bodyweight status was defined according to the following categories: obese (BMI ≥ 30 kg/m^2^), overweight (BMI ≥ 25 to < 30 kg/m^2^), normal weight (BMI ≥ 18.5 to < 25 kg/m^2^), and underweight (BMI < 18.5 kg/m^2^). A *p* < 0.05 was considered to be statistically significant. Statistical analysis was performed using the Statistical Package for the Social Sciences (SPSS) version 26.0 (IBM, Chicago, IL, USA).

## Results

### Demographic Characteristics

A total of 2,507 participants completed the online questionnaire. The demographic breakdown of the surveyed participants is presented in Table 1. Most of the participants completed the survey in the English language (53.5%) followed by Arabic (39.9%) and French (6.6%).

**Table 1:** Demographic breakdown of surveyed participants (*n* = 2,507).

**Characteristics**	***n***	**%**
**Age (years)**		
18–25	1,301	51.9
26–35	641	25.6
36–45	308	12.3
>46	257	10.2
**Gender**		
Male	270	27.0
Female	1,830	73.0
**Marital status**		
Married	829	33.3
Single	1,612	64.3
Divorced/Widowed	66	2.6
**Number of children in the family**		
None	1,793	71.5
1–2 children	471	18.8
≥ 3 children	243	9.7
**Education level**		
Intermediate or less	60	2.4
High school/Diploma	802	32.0
University degree	1,645	65.6
**Employment status**		
Employee	972	38.7
Self-employed	699	27.9
Unemployed	573	22.9
Student	240	9.6
Retired	23	0.9
**Currently, work/study from home**		
Yes	1,409	56.2
No/Not applicable	1,098	43.8
**Governorate of residence**		
Beirut	408	16.3
Mount Lebanon	928	37.0
North	472	18.8
South	322	12.8
Nabatiye	56	2.2
Beqaa	321	12.8
**Current weight status** ^**a**^		
Underweight	162	6.5
Normal weight	1,343	53.6
Overweight	630	25.1
Obesity	372	14.8
**Bodyweight change during the pandemic**		
Lost weight	724	28.9
Gained weight	823	32.8
Maintained weight	853	34.0
Do not know	107	4.3
**The general state of health in the past 3 months**		
Excellent	327	13.0
Very good	820	32.7
Good	927	37.0
Fair	393	15.7
Poor	40	1.6

a *Based on BMI classification*.

Most surveyed individuals were females (73%), aged 18–25 years (51.9%), were single (64.3%), did not have children (71.5%), completed a university degree (65.6%), and were employed (38.7%). Also, during the lockdown, most of the individuals were working or studying from home (56.2%). Participants joined from six governorates of Lebanon (Beirut, Mount Lebanon, North, South, Nabatiyeh, and Beqaa) and the sample distribution from different governorates was representative of the population distribution in Lebanon. The largest proportion of partakers were from Mount Lebanon (37.0%), North (18.8%), Beirut (16.3%), and South (12.8%).

Regarding their health and weight status, most participants had normal BMI (53.6%), while 25.1% were overweight and 14.8% were obese. Weight was maintained in 34.0% of the individuals whereas 32.8% of them claimed weight gain and 28.9% reported weight loss during the pandemic. When asked about their general health status during the past 3 months, most of the individuals reported good health (37.0%) and very good health (32.7%). Only 1.6% specified a poor state of health.

### Source of Health and Nutrition Information

Responses about the most common source of health and nutrition updates during the COVID-19 pandemic are presented in Table 2. The most common source of information for both health and nutrition updates and recommendations were social media platforms (65.4 and 65.8%) respectively, followed by healthcare professionals (45.2 and 48.3%) respectively. Only 36.6 and 27.7% of participants selected local and international health authorities as a source of information for health and nutrition updates, respectively.

**Table 2:** Sources of health and nutrition information during COVID-19 pandemic (*n* = 2,507).

**Sources of information^**a**^**	**Health-related information** ***n* (%)**	**Nutrition-related information** ***n* (%)**
Health authorities	911 (36.6)	683 (27.2)
Social media	1,639 (65.4)	1,649 (65.8)
Professionals	1,134 (45.2)	1,212 (48.3)
Television	620 (24.7)	447 (17.8)
Newspapers	94 (3.7)	74 (3.0)
Friends	866 (34.5)	886 (35.3)

a *The total number of responses is greater than the total number of participants and the percent of cases displayed since multiple responses were allowed for this question*.

### Eating Habits

The results on the eating habits of the study participants pre-and during the COVID-19 pandemic are presented in Table 3. There was a significant increase in the percentage of participants eating mostly homemade meals during COVID-19 (95.5%) compared to pre-COVID-19 (83.3%) (*p* < 0.001).

**Table 3:** Eating habits pre-and during COVID-19 pandemic (*n* = 2,507).

**Variables**	**Pre-COVID-19** ***n* (%)**	**During COVID-19** ***n* (%)**	***p*-value (2-sided)**
**Most consumed meals during the week** ^**a**^			
Homemade	2,089 (83.3)	2,393 (95.5)	<0.001
Frozen ready-to-eat	181 (7.2)	141 (5.6)	0.005
Fast food	815 (32.5)	279 (11.1)	<0.001
Restaurants	656 (26.2)	135 (5.4)	<0.001
Healthy food from restaurants	226 (9.0)	104 (4.1)	<0.001
**Number of meals eaten per day**			
1–2 meals	957 (38.2)	675 (26.9)	<0.001
3–4 meals	1,427 (56.9)	1,523 (60.7)	0.003
≥5 meals	123 (4.9)	309 (12.3)	<0.001
**Eating breakfast on most days of the week**			
Yes	1,598 (63.7)	1,708 (68.1)	<0.001
No	909 (36.3)	799 (31.9)	
**Skipping meals**			
Yes	1,554 (62.0)	100 (40.2)	<0.001
No	953 (38.0)	1,498 (59.8)	
**Reasons for skipping meals (If the answer was yes)** ^**a**^			
Reduced food	224 (14.6)	198 (20.0)	<0.001
Lack of time	1,034 (67.2)	320 (32.3)	<0.001
Lose weight	268 (17.4)	213 (21.5)	0.001
Lack of appetite	428 (27.8)	408 (41.2)	<0.001
Fasting	222 (14.4)	179 (18.1)	0.509
**Amount of water consumed per day**			
1–4 cups	1,151 (45.9)	914 (36.5)	<0.001
5–7 cups	811 (32.3)	912 (36.4)	<0.001
≥8 cups	545 (21.7)	681 (27.2)	<0.001

a *The total number of responses is greater than the total number of participants and the percent of cases displayed since multiple responses were allowed for this question. The p values indicate the statistical significance of the McNemar test*.

Moreover, the percentage of surveyed individuals consuming five or more meals per day increased significantly from 4.9% pre-COVID-19 to 12.3% during COVID-19 (*p* < 0.001). Also, there was a decrease in the percentage of individuals skipping breakfast on most days of the week from 36.3% pre-COVID-19 to 31.9% during the pandemic (*p* < 0.001). Similarly, the percentage of participants skipping meals during the day decreased from 62.0 to 59.8% (*p* < 0.001). Before the pandemic, the main reason for skipping meals was lack of time (67.2%), however during the pandemic lack of appetite (41.2%) was the main reason to skip meals. While there was an increase in the number of meals during the pandemic, water recommendation was not met by 72.9% of participants who reported drinking less than eight cups of water per day during the outbreak. However, this percentage was even higher before the pandemic (78.3%).

The frequency of consuming certain foods during the COVID-19 pandemic is presented in Table 4. Overall, 44.7% of the participants did not eat fruits every day and 35.3% did not eat vegetables daily. On the other hand, 28.0% reported consuming sweets or desserts at least once per day and 30.9% consumed salty snacks (nuts, crackers, chips) every day. Also, 60.7% of the individuals reported drinking coffee or tea at least once per day. Sweetened drinks were more popular compared to energy drinks among the participants, as 24.7% stated drinking sweetened drinks at least once per day and only 4.8% claimed consuming energy drinks on daily basis.

**Table 4:** The frequency of consumption of certain foods during the COVID-19 pandemic (*n* = 2,507).

**Food items**	**≥4 times/day**	**2–3 times/day**	**Once/day**	**1–4 times/week**	**Never**
	***n*** **(%)**				
Fruits	69 (2.8)	534 (21.3)	783 (31.2)	1,012 (40.4)	109 (4.3)
Vegetables	113 (4.5)	670 (26.7)	839 (33.5)	827 (33.0)	58 (2.3)
Milk and milk products	35 (1.4)	314 (12.5)	918 (36.6)	1,002 (40.0)	238 (9.5)
Meat/fish/chicken	34 (1.4)	202 (8.1)	936 (37.3)	1,258 (50.2)	77 (3.1)
Bread/rice/pasta	85 (3.4)	538 (21.5)	787 (31.4)	1,027 (41.0)	70 (2.8)
Sweets/desserts	51 (2.0)	251 (10.0)	701 (28.0)	1,200 (47.9)	304 (12.1)
Salty nuts/crackers/chips	36 (1.4)	160 (6.4)	579 (23.1)	1,345 (53.6)	387 (15.4)
Coffee/tea	156 (6.2)	580 (23.1)	786 (31.4)	697 (27.8)	288 (11.5)
Sweetened drinks	45 (1.8)	166 (6.6)	409 (16.3)	1,022 (40.8)	865 (34.5)
Energy drinks	12 (0.5)	28 (1.1)	79 (3.2)	329 (13.1)	2,059 (82.1)

### Shopping

Fortunately, hygienic practices were adopted by the majority of the surveyed individuals, as shown in Table 5. Only 7.7% of the participants reported not cleaning/sanitizing groceries before storage. Besides, 60.8% of the participants addressed preparing a shopping list before grocery shopping. Moreover, most of the participants conveyed stocking up on foods during the pandemic (60.6%). Online shopping was less common among the participants since 28.8% of them claimed to order groceries online. The main reasons for ordering groceries online were staying safe, saving time, and convenience (68.5, 50.1, and 49.3% respectively).

**Table 5:** Shopping practices during COVID-19 pandemic (*n* = 2,507).

**Variables**	***n* (%)**
**Prepare shopping list before grocery shopping**	
Yes	1,525 (60.8)
No	982 (39.2)
**Started stocking up on foods**	
Yes	1,518 (60.6)
No	556 (22.2)
Already stocking up	433 (17.3)
**Ordering groceries online**	
Yes	721 (28.8)
No	1,786 (71.2)
**If yes, why do you prefer ordering online** ^**a**^	
Stay safe	490 (68.5)
Save time	358 (50.1)
Choose only the needed items	197 (27.6)
Avoid crowds	347 (48.5)
Easy	354 (49.5)
**Reading food labels before purchasing**	
Yes	1,381 (55.1)
No	299 (11.9)
Sometimes	827 (33.0)
**Sanitizing/ cleaning groceries before storage**	
Yes	188 (75.3)
No	193 (7.7)
Sometimes	427 (17.0)

a *The total number of responses is greater than the total number of participants and the percent of cases displayed since multiple responses were allowed for this question*.

### Physical Activity

**Figure 1:**
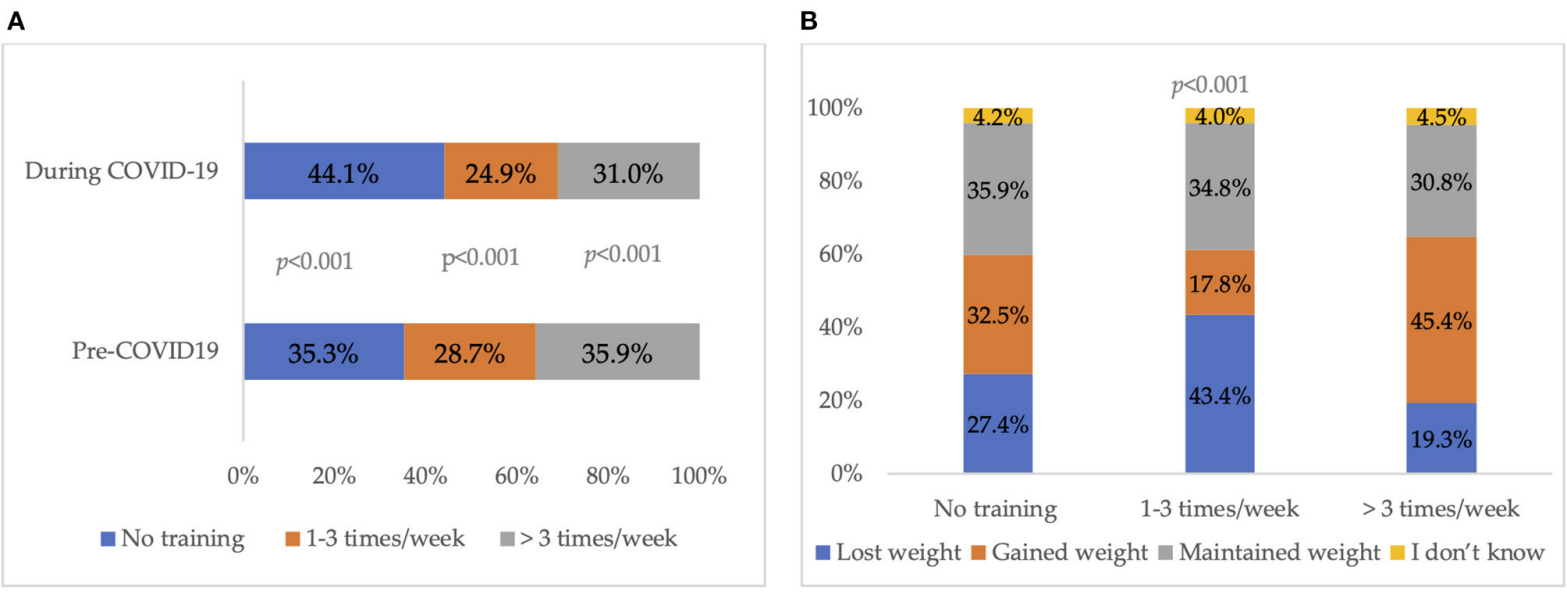
**(A)** Frequency of physical activity pre-and during COVID-19 pandemic. The *p* values indicate the statistical significance of the McNemar test. **(B)** Physical activity during the COVID-19 pandemic with the perceived change in weight. The *p* values indicate the statistical significance of the Chi-Square test.

Figure 1A shows 35.3% of the individuals reported not exercising before the outbreak and the percentage increased to 44.1% during the pandemic (*p* < 0.001). When asked about the reasons for motivation to perform physical activity, the most common answers were to be healthy (77.7%), to lose or maintain weight (63.2%), and to release stress (58.9%). Figure 1B displays that the frequency of performing physical activity during the COVID-19 pandemic was significantly associated with the perceived change in weight (*p* < 0.001). Unexpectedly, 45.4% of the individuals who exercised more than three times per week reported weight gain during the pandemic.

As shown in Table 6, 34.8% of participants spent more than 5 h in front of the screen for study or work purposes during COVID-19 compared to 19.9% before the pandemic (*p* < 0.001). Spending screen time for entertainment purposes increased significantly from 11.2% before the lockdown to 38.8% during the COVID-19 lockdown (*p* < 0.001). Also, spending time doing household chores every day increased significantly from 22.1% before the pandemic to 31.9% during the pandemic (*p* < 0.001).

**Table 6:** Daily activities pre-and during COVID-19 pandemic (*n* = 2,507).

**Variables**	**Pre-COVID-19** ***n* (%)**	**During COVID-19 *n* (%)**	***p*-value (2-sided)**
**Doing household chores**			
Never	1,077 (43.0)	750 (29.9)	<0.001
1–3 times/week	724 (28.9)	723 (28.8)	1
4–5 times/week	151 (6.0)	234 (9.3)	<0.001
Everyday	555 (22.1)	800 (31.9)	<0.001
**Screen time for study or work**			
None	609 (24.3)	501 (20.0)	<0.001
1–2 h/day	878 (35.0)	501 (20.0)	<0.001
3–5 h/day	521 (20.8)	633 (25.2)	<0.001
>5 h/day	499 (19.9)	872 (34.8)	<0.001
**Screen time for entertainment**			
<30 min/day	348 (13.9)	168 (6.7)	<0.001
1–2 h/day	1,200 (47.9)	496 (19.8)	<0.001
3–5 h/day	677 (27.0)	871 (34.7)	<0.001
>5 h/day	282 (11.2)	972 (38.8)	<0.001

*The p values indicate the statistical significance of the McNemar test*.

### Stress

**Figure 2:**
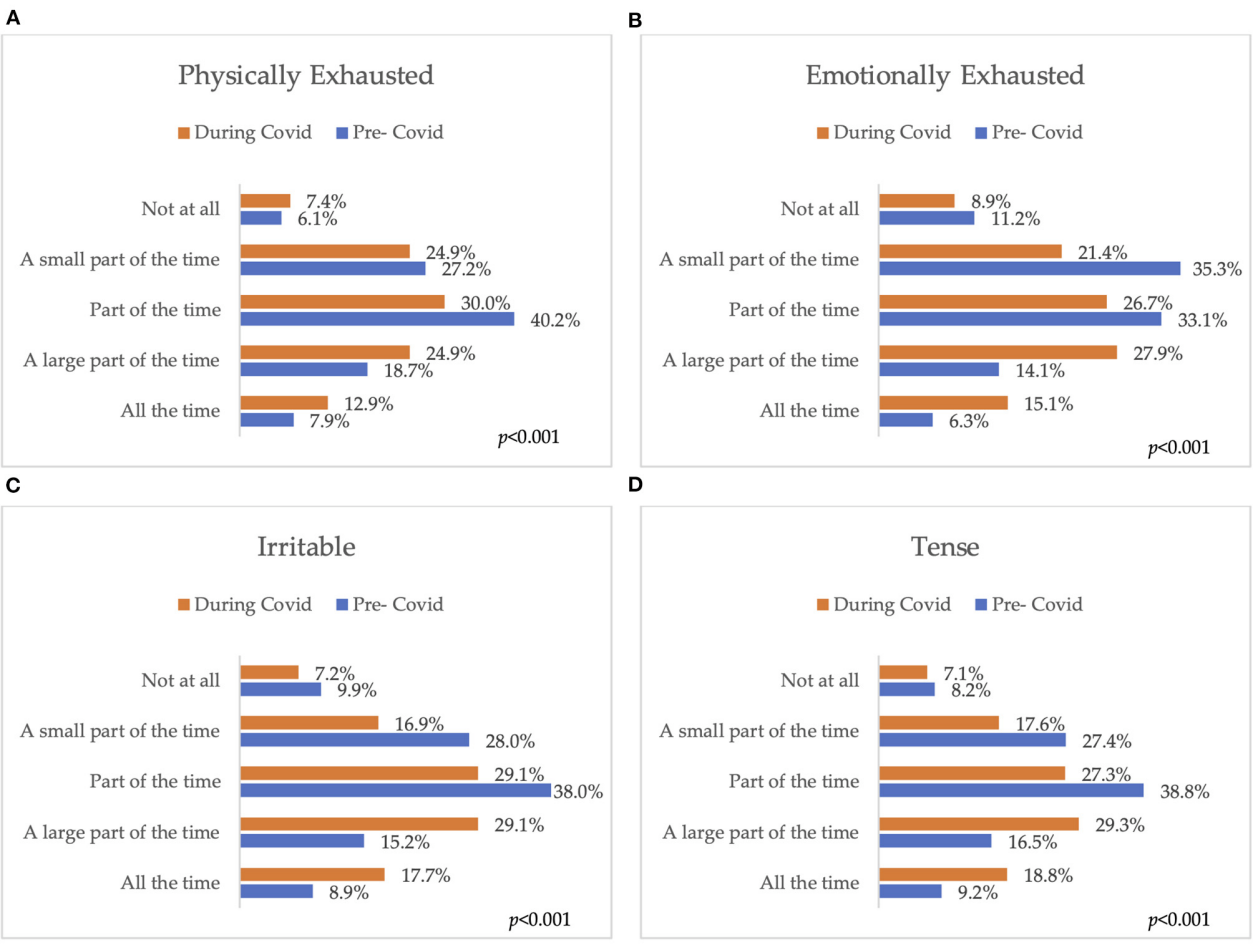
Stress and irritability pre-and during the COVID-19 pandemic **(A)** Physical exhaustion; **(B)** Emotional exhaustion; **(C)** Irritability; **(D)** Tension. The *p* values indicate the statistical significance of the McNemar test.

Figure 2 displays the frequency of the individuals experiencing different forms of stress before and during the pandemic. Being physically exhausted, a large part of the time before the pandemic (18.7%) increased to 24.9% during the pandemic (*p* < 0.001). Also, emotional exhaustion, a large part of the time increased from 14.1% before the pandemic to 27.9% during the pandemic (*p* < 0.001). Moreover, participants claimed to be more irritable and tense most of the time during the pandemic (from 15.2–17.7% and from 16.5–29.3% respectively, *p* < 0.001).

### Sleep

Results in Table 7 show a significant increase in the percentage of participants who reported sleeping more than 9 h from 3.4–20.3% (*p* < 0.001). However, a significantly higher percentage of participants classified their sleep quality as poor during the pandemic (29.6%) compared to before the pandemic (22.1%) (*p* < 0.001). Besides, a significantly higher percentage of participants claimed to experience sleep disturbances during the pandemic (89.5%) compared to before (66.8%) (*p* < 0.001). Thus, a significantly higher percentage of participants reported feeling lazy during the pandemic (30.5%) compared to pre-COVID-19 (*p* < 0.001).

**Table 7:** Sleep pre-and during COVID-19 pandemic (*n* = 2,507).

**Variables**	**Pre-COVID-19** ***n* (%)**	**During COVID-19 *n* (%)**	***P*-value (2-sided)**
**Hours of sleep per night**			
<7 h	1,291 (51.5)	783 (31.2)	<0.001
7–9 h	1,131 (45.1)	1,214 (48.4)	<0.001
>9 h	85 (3.4)	510 (20.3)	<0.001
**How would you rate your sleep quality**			
Very good	477 (19.0)	621 (24.8)	<0.001
Good	1,475 (58.8)	1,145 (45.7)	<0.001
Poor	555 (22.1)	741 (29.6)	<0.001
**Did you experience any of the following** ^**a**^			
Slept badly and restlessly	629 (25.1)	644 (25.7)	0.591
Hard to go to sleep	605 (24.1)	946 (37.7)	<0.001
Woken up too early and have not been able to get back to sleep	520 (20.7)	489 (19.5)	0.197
Woken up several times and found it difficult to get back to sleep	441 (17.6)	654 (26.1)	<0.001
None	1,192 (47.5)	946 (37.7)	<0.001
**Describe your energy level**			
Energized	1,078 (43.0)	566 (22.6)	<0.001
Neutral	1,320 (52.7)	1,176 (46.9)	<0.001
Lazy	109 (4.3)	765 (30.5)	<0.001

a *The total number of responses is greater than the total number of participants and the percent of cases displayed since multiple responses were allowed for this question. The p values indicate the statistical significance of the McNemar test*.

## Discussion

This study presents the findings of a web-based survey collected in six governorates of Lebanon and in three languages. The survey revealed an increase in body weight, consumption of homemade meals, number of meals per day, sedentary time, stress, and sleeping hours during the COVID-19 pandemic compared to before the pandemic. The findings also suggested a decrease in physical activity, sleep quality, and energy level. Additionally, data on dietary habits revealed low intake of water, fruit, and vegetables, and high consumption of sweets, salty snacks, and caffeinated and sweetened beverages during the pandemic.

The results of the current study reveal expected unfavorable changes in the dietary habits of the surveyed participants such as an increase in the frequency of meals consumed per day. On the other hand, some favorable changes were reported like higher intake of water and consumption of homemade meals. These findings concur with the recent French NutriNet-Sante Cohort Study which clusters participants into two main groups; group 1 presented unfavorable nutritional trends during the pandemic (increased snacking, decreased consumption of fresh food products, and increased consumption of sweets and biscuits), while group 2 displayed opposite trends (increased home-made cooking) (36). While more homemade meals were consumed in the current study, more non-nutritious foods were chosen (sweets, salty snacks, and sweetened drinks), as well as the meals were more frequently consumed. These data could explain the reported weight gain as perceived by the study sample. Similarly, results from Italy, United States, Poland, France, and Kuwait revealed weight gain during the COVID-19 pandemic along with an increase in caloric intake (19, 24, 36–38). Moreover, results of the ECLB-COVID19 international online survey indicated higher consumption of unhealthy foods, more frequent snacking, and a higher number of meals consumed per day during the pandemic (23). Zachary et al. suggested that increased time spent at home may provoke additional eating in response to non-nutritive cues, along with insufficient sleep, frequent snacking, lack of dietary restraint, emotional eating, and reduced physical activity (39). A systematic review revealed that confinement tended to exacerbate pre-existing weight status, with overweight/obese individuals more destined to add more weight and underweight individuals more likely to report losing weight (40). The review identified risk factors for weight gain increased consumption of junk food, snacking and declined intake of fresh fruits and vegetables, physical activity, and altered sleep (40). The results of the current study showed an obesity prevalence of 14.8%, lower than the previous cross-sectional survey among the Lebanese adults where the obesity rate was reported to be 22% (41).

According to the WHO, nutrition transition in Lebanon is still considered at an early stage, characterized by moderate levels of overweight and obesity, besides moderate levels of undernutrition and micronutrient deficiencies among specific subpopulations and age groups (42). Among the unfavorable food patterns examined, was reduced intake of fruits and vegetables and higher intake of denser food choices such as sweets, salty snacks, and sweetened drinks. Moreover, eating energy-dense, palatable foods could be linked to emotional eating where people tend to consume such foods as a coping mechanism to regulate pandemic related negative emotions, such as depression, anxiety, and stress (43). The reduction in consumption of fruits can be attributed to a significant increase in the prices of fruits and vegetables (161 and 53%) respectively (29). Dense food choices are known to be rich in unhealthy saturated fats, trans fats, and added sugars. It is well documented that antioxidants and phytochemicals are the foremost food components in boosting immunity (44). Thus, low consumption of these specific dietary components may trigger the release of pro-inflammatory markers such as cytokines interleukin-6 (IL-6), IL-1β, and tumor necrosis factor (TNF)-alpha (45). Consequently, lack of these nutrients may also prompt the development and advancement of COVID-19 and its severe complications (46). Besides, a high intake of added sugar and sugar-sweetened beverages (SSBs) might elevate the level of circulating inflammatory proteins and chronic inflammation and may contribute to the onset of type 2 diabetes (47).

Another observation in this study was decreased dairy products consumption, a food group rich in calcium and vitamin D. Enough evidence indicates the importance of vitamin D in reducing the risk of acute respiratory tract infections from COVID-19 and suppressing the risk of inflammatory cytokine production (48). This beneficial effect of vitamin D can be attributed to a 116% increase in the prices of dairy products (29). A study conducted in Lebanon investigating the nutritional implications of the 2004/2008 food prices increase, indicated a significant drop in macro/micronutrient intakes which are considered key indicators for malnutrition risk (49).

Linking these results to the existing studies of the Lebanese dietary habits, the current dietary trend is experiencing a shift in food consumption away from the traditional cuisine, or what is known as the Mediterranean Diet (MD) (50). A diet that heavily relies on vegetarian recipes, legumes, cereals, an abundance of fruits and nuts, as well as the consumption of olive oil as the main fat. Over the past decades, numerous epidemiological studies have investigated the influence of adherence to the MD on health outcomes, ultimately showing protective associations with a host of non-communicable diseases ranging from cardiovascular disease to cancer (51, 52). While a westernized diet characterized by an ample supply of saturated fat, *trans* fat components, simple sugars, and low levels of antioxidants, fibers, and unsaturated fats, impacts the immune system against various infection factors.

Taken all these factors aforesaid along with the fragile health care system, contributed to the alarming rates of COVID-19 infections and high mortality rate. Given the country's extended periods of political upheaval and considerable governance, may have further strained the weak economic development of the country and affected the affordability of purchasing healthy foods. Our results highlight the pressing need for public interventions to combat and prevent nutrients deficiencies, through reverting traditional cuisine, encouraging home plantation and good food choices.

The results of this survey though revealed improved water intake per day, also found that in contrast to the guidance of the World Health Organization (53), the majority of participants reported drinking less than eight cups of water per day during the COVID-19 pandemic. Likewise, surveyed participants in the Middle East region, United Arab Emirates, and Italy were not meeting the recommended water intake during the pandemic (17, 19, 21).

The COVID-19 pandemic has dramatically impacted lifestyle activities globally. A decrease in physical activity and an increase in sedentary behaviors during the coronavirus pandemic were observed in the current study. These results are in agreement with other studies indicating that the COVID-19 pandemic had reduced physical activity worldwide (17, 19, 21, 23, 24, 36–38). The current study assessing pre-COVID physical activity rate was similar to a previous study among the Lebanese (41).

Surprisingly, individuals in the current sample who exercised more than three times per week reported weight gain. Another sedentary behavior observed among the sample of participants connected with prolonged time sitting at home and confinement, is the increased screen time spent for entertainment. It is worth noting that this sedentary behavior is associated with increased risks of depression (54) and suppressed immune competency (55). Therefore, lower inactivity combined with frequent snacking and energy surplus has led to weight gain as perceived by 32.8% of the population. If these adverse trends concerning physical inactivity and unhealthy dietary habits are not intervened, they might not only contribute to weight gain but will also increase the risk of cardiovascular disease in the future (56). Therefore, awareness programs about the importance of healthy lifestyle modifications are essential during such unprecedented times (57).

Higher levels of stress, depression, anxiety, aggression, and fear of infection have been witnessed during the COVID-19 pandemic among individuals (58–61). Data from the current study revealed a significant increase in the percentage of participants experiencing exhaustion, irritability, and tension during the pandemic. Similarly in the United Arab Emirates, participants felt horrified, apprehensive, or helpless due to the COVID-19 pandemic (62). In Lebanon, citizens reported an increase in Post-traumatic Stress Disorder (PTSD) symptomatology during the second week of the COVID-19 lockdown (63) and higher fear of COVID-19, anger, and anxiety in almost half of the participants (64). It was also suggested that fear of COVID-19 was correlated with more eating restraint, weight, and shape concerns in the whole sample (64). This psychological distress encountered among participants during the quarantine ought to be considered in relation to multiple factors. In addition to the forced absence of social life and health anxiety, a low economy had influenced young people's mental well-being. They are worried about their future and uncertainty of employment stability. This calls for the governmental and local social welfare associations for empowering society to handle outbreaks by, motivating young people to engage in volunteer works, social support correctness, dissemination of accurate health education, and disease transmission (65).

Good hygienic practices during shopping were adopted by the majority of the surveyed individuals in the current study. This is in agreement with findings by Faour-Klingbeil et al. where 83.1% of participants in Lebanon reported that they disinfect always or often food packaging before storing them away at home, which was higher than reported in Jordan and Tunisia (66). Social media played a major role in this regard as indicated by Faour-Klingbeil et al. (67).

In addition, our study revealed that 55.1% read food labels before purchasing, 33% do it sometimes and 11.9% do not read them. Hassan and Dimassi reported that in Lebanon, 29.1% of consumers read food labels every time before buying a product, while 55.2% do it sometimes and 15.7% never do it (68). Our findings thus indicate that reading labels became more evident since the COVID-19 pandemic emerged. This can be attributed to increasing awareness regarding healthy behavior among the general public in light of the pandemic.

The current study describes unfavorable eating behaviors, lifestyle changes, and psychological distress associated with confinement during the COVID-19 outbreak, on a sample of the Lebanese population. Meanwhile, several prominent challenges faced Lebanon's population, worsened by the economic slowdown due to the spread of the COVID-19 pandemic such as the steep inflation of prices, low economy, and fragile health care system (64). All of these factors might inflict negative health consequences and impose a further economic burden. The findings of this study highlight the need for wider social programs, non-governmental agencies, and public health interventions through traditional and social media to revitalize the traditional healthy cuisine of the nation.

Although this study provides an insight into the impact of the pandemic on dietary patterns and lifestyle, there are few limitations to be underlined. The use of the snowball convenience sampling method, self-reported survey, the cross-sectional study design, and the higher ratio of female respondents might all have impacted the generalizability of the results. Also, infection with COVID-19 was not examined in the study. However, despite being an online survey in a country where not everyone has smartphones that nor are connected to the internet, the use of such a tool allowed data collection during the COVID-19 pandemic restriction from different parts of Lebanon and covered a good sample number. It also ensured the anonymity of the participants, hence reduced the social desirability bias.

## Data Availability Statement

The original contributions presented in the study are included in the article/Supplementary Material, further inquiries can be directed to the corresponding author/s.

## Ethics Statement

The studies involving human participants were reviewed and approved by the Research Ethics Committee at the University of Sharjah (REC-20-04-25-02) and the Social Sciences Research Ethics Committee at United Arab of Emirates University (ERS_2020_6106). The patients/participants provided their written informed consent to participate in this study.

## Author Contributions

LC, TO, and ASA: conceptualization. LC, MH, MM, SS, TO, and ASA: methodology. LC, MM, TO, AA, and ASA: validation. LC, MM, and HHasa: formal analysis. LC, MH, MM, HHass, AA, LS, AJ, HHasa, DA, SS, RA, TO, and ASA: investigation. LC, MM, SS, and ASA: writing—original draft preparation. LC, MH, MM, HHass, AA, LS, AJ, HHasa, DA, SS, RA, TO, and ASA: writing—review and editing. All authors have read and agreed to the published version of the manuscript.

## Conflict of Interest

The authors declare that the research was conducted in the absence of any commercial or financial relationships that could be construed as a potential conflict of interest.

## Publisher's Note

All claims expressed in this article are solely those of the authors and do not necessarily represent those of their affiliated organizations, or those of the publisher, the editors and the reviewers. Any product that may be evaluated in this article, or claim that may be made by its manufacturer, is not guaranteed or endorsed by the publisher.
